# A deletion mutation in bovine *SLC4A2 *is associated with osteopetrosis in Red Angus cattle

**DOI:** 10.1186/1471-2164-11-337

**Published:** 2010-05-27

**Authors:** Stacey N Meyers, Tara G McDaneld, Shannon L Swist, Brandy M Marron, David J Steffen, Donal O'Toole, Jeffrey R O'Connell, Jonathan E Beever, Tad S Sonstegard, Timothy PL Smith

**Affiliations:** 1 Laboratory of Molecular Genetics, Department of Animal Sciences, University of Illinois at Urbana-Champaign, Urbana, IL 61801, USA; 2 U.S. Meat Animal Research Center, USDA, ARS, Clay Center, Nebraska, USA; 3 Department of Veterinary Sciences, University of Wyoming, Laramie, WY 82070, USA; 4 Department of Veterinary and Biomedical Sciences, Institute of Agriculture and Natural Resources, University of Nebraska, Lincoln, NE 68583, USA; 5 University of Maryland School of Medicine, Baltimore, MD 21201, USA; 6 Bovine Functional Genomics Laboratory, United States Department of Agriculture, Agricultural Research Service, Beltsville, Maryland, USA

## Abstract

**Background:**

Osteopetrosis is a skeletal disorder of humans and animals characterized by the formation of overly dense bones, resulting from a deficiency in the number and/or function of bone-resorbing osteoclast cells. In cattle, osteopetrosis can either be induced during gestation by viral infection of the dam, or inherited as a recessive defect. Genetically affected calves are typically aborted late in gestation, display skull deformities and exhibit a marked reduction of osteoclasts. Although mutations in several genes are associated with osteopetrosis in humans and mice, the genetic basis of the cattle disorder was previously unknown.

**Results:**

We have conducted a whole-genome association analysis to identify the mutation responsible for inherited osteopetrosis in Red Angus cattle. Analysis of >54,000 SNP genotypes for each of seven affected calves and nine control animals localized the defective gene to the telomeric end of bovine chromosome 4 (BTA4). Homozygosity analysis refined the interval to a 3.4-Mb region containing the *SLC4A2 *gene, encoding an anion exchanger protein necessary for proper osteoclast function. Examination of *SLC4A2 *from normal and affected animals revealed a ~2.8-kb deletion mutation in affected calves that encompasses exon 2 and nearly half of exon 3, predicted to prevent normal protein function. Analysis of RNA from a proven heterozygous individual confirmed the presence of transcripts lacking exons 2 and 3, in addition to normal transcripts. Genotyping of additional animals demonstrated complete concordance of the homozygous deletion genotype with the osteopetrosis phenotype. Histological examination of affected tissues revealed scarce, morphologically abnormal osteoclasts displaying evidence of apoptosis.

**Conclusions:**

These results indicate that a deletion mutation within bovine *SLC4A2 *is associated with osteopetrosis in Red Angus cattle. Loss of SLC4A2 function appears to induce premature cell death, and likely results in cytoplasmic alkalinization of osteoclasts which, in turn, may disrupt acidification of resorption lacunae.

## Background

Recessive genetic diseases have occasionally emerged throughout the history of domestic cattle populations. In part, this can be attributed to selective breeding practices involving the extensive use of particular individuals or pedigrees with demonstrated genetic merit. These practices can potentially lead to a reduction in the effective population size and a subsequent increase in homozygosity, thus allowing for the expression of recessive defect phenotypes. The management of such disorders requires the identification and culling of carrier individuals within populations. Traditionally, when the genetic basis of a recessive disorder is not known, identification of carriers has been achieved through progeny testing or practicing "pedigree prejudice", i.e., avoiding use of individuals of a pedigree suspected to contain carriers. Unfortunately, progeny testing is time-consuming and costly, and the indiscriminate culling of pedigrees potentially results in the loss of valuable germplasm. The development and use of DNA-based tools for identifying carriers can alleviate these issues, providing the impetus for mapping disease loci. Tremendous advances in bovine genome characterization over the last two decades, including the completion of the bovine genome sequence [1] and the development of thousands of molecular markers, have provided the necessary resources for the efficient elucidation of the genetic mutations underlying heritable disorders.

Osteopetrosis is a bone disorder known to affect humans and other animals; in cattle, the disease can be virally-induced [2,3], yet is typically inherited as a recessive defect [4]. It has been observed in several breeds, including Angus, Red Angus, Hereford, Simmental and Holstein [3-11]. In the 1960s, the number of reported cases of osteopetrosis increased in frequency; during the six-year period from 1967 to 1973, 123 affected Angus calves were identified in the United States and Canada [7]. Through the use of traditional progeny testing and pedigree prejudice disease management methods, the occurrence of osteopetrosis was moderated. However, there has been a recent recurrence of the disease in the Red Angus cattle. Laboratory-confirmed cases were identified in herds in Wyoming, Nebraska, Missouri, Kansas and Saskatchewan [3,11], renewing economic and animal welfare concerns among breeders. Moreover, the cause for concern is exacerbated by the current use of reproductive technologies such as artificial insemination and embryo transfer that can rapidly disseminate deleterious alleles.

**Figure 1:**
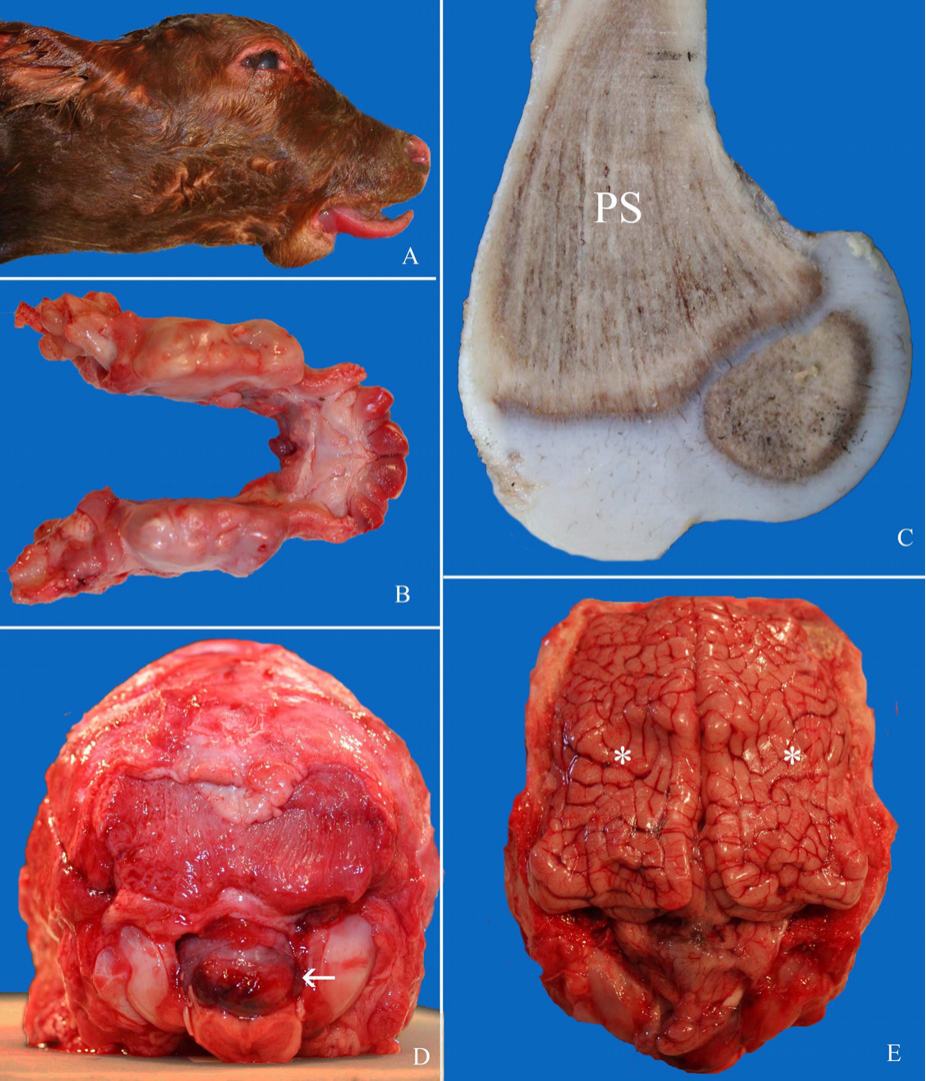
**Phenotype of congenital osteopetrosis in affected Red Angus calves**. **A**. Head of a late-term stillborn calf with inferior brachygnathia, short flattened skull and protruding tongue. **B**. Impacted unerupted molar (M) and incisor teeth (I) in mandibles of an affected calf. **C**. Posterior aspect of skull with partial herniation of cerebellum through foramen magnum (arrow). **D**. Distal aspect of a sagittally sectioned femur with a triangle of primary spongiosa (PS) resting on the distal growth plate and extending into the diaphysis. The normal marrow cavity is effaced. **E**. Marked compression of cerebral hemispheres (asterisk) due to excessive thickness of calvarium. The lesions shown in C and E (cerebellar herniation and compression of cerebral hemispheres), combined with mineralization within the brain, are likely responsible for death of calves *in utero *with stillbirth.

The defining characteristics of osteopetrosis (also known as "marble bone disease") are the defective activity of osteoclasts, large multinucleated cells that resorb bone, and the resulting accumulation of primary spongiosa in marrow cavities (reviewed in, e.g., [12-14]). This results in the formation of extremely dense, fragile bones. Various secondary pathological conditions are also observed; in cattle, affected calves are typically stillborn, slightly premature (250-275 days of gestation), and often display features including small body size, flat skull, impacted molars, shortened mandible (brachygnathia inferior), and protruding tongue ([3-11]; Figure 1). The osteopetrosis phenotype indicates a defect in normal bone development and remodeling, and more specifically, an imbalanced interplay between bone-forming osteoblasts and bone-resorbing osteoclasts (reviewed in, e.g., [12-14]). Osteopetrosis is known to result from a deficiency in the number and/or function of osteoclasts; indeed, in cases of inherited osteopetrosis in Angus cattle, few osteoclasts have been observed [6,9,10], and any existing osteoclasts lack surrounding zones of resorption [10].

Normally, differentiation of osteoclasts is induced by the binding of a ligand on the surface of osteoblasts, to its receptor on the surface of mononuclear osteoclast precursors (reviewed in, e.g., [12-14]). Following differentiation, osteoclasts resorb bone through a three step process, involving 1) cytoskeletal organization and the formation of a ruffled border membrane, 2) acidification of the resorption pit formed between the osteoclast and the bone surface (Howship's lacuna), which serves to demineralize inorganic bone material, and 3) proteolysis of the organic matrix. The acidification step is achieved through an active process in which protons produced in the osteoclast by the action of carbonic anhydrase II (CA2) are pumped across the ruffled border membrane by a multi-subunit, vacuolar ATPase. Net charge in the lacuna is neutralized by concurrent movement of chloride ions from the osteoclast through a channel composed of the CLCN7 and OSTM1 proteins. To complete the biochemical cycle and prevent toxic buildup in the osteoclast of carbonate ions produced by CA2, an anion exchanger on the external cell surface, SLC4A2, exports carbonate ions and imports chloride ions. The subsequent proteolysis step is primarily mediated by the enzyme cathepsin K (CTSK).

In humans, mutations in at least ten different genes are known to cause osteopetrosis or osteopetrosis-like syndromes [14], accounting for approximately 70% of osteopetrosis cases [15]. The molecular basis of the remaining 30% is unknown. Mutated genes include *TNFSF11 *and *TNFRSF11A*, encoding the ligand/receptor pair involved in osteoclast differentiation; *ITGB3*, encoding the β3 integrin involved in cytoskeletal organization; *TCIRG1*, encoding the a3 subunit of the v-ATPase; *CA2*, encoding carbonic anhydrase II; *CLCN7 *and *OSTM1*, encoding the chloride channel and a channel stabilizing protein; and *CTSK*, encoding cathepsin K. The remaining two genes, *PLEKHM1 *("pleckstrin homology domain containing, family M member 1") and *IKBKG *("inhibitor of kappa light polypeptide gene enhancer in B-cells, kinase gamma"), are less well characterized.

In this study, we describe the clinical presentation and histopathology of osteopetrosis in Red Angus cattle, the mapping of the disease locus and the identification of a ~2.8-kb deletion mutation disrupting the *SLC4A2 *gene. We further demonstrate complete concordance between the homozygous deletion genotype and the osteopetrosis phenotype, and confirm the presence of both normal and mutant *SLC4A2 *transcripts in a known carrier of the disease.

## Results

### Clinical presentation and histopathology

Thirteen calves displaying the osteopetrosis phenotype were acquired throughout the course of this study; all calves exhibited typical gross lesions ([3-11]; Figure 1). Tissues of affected calves were examined for evidence of transplacental infection with bovine viral diarrhea virus (BVDV), a recognized infectious cause of osteopetrosis [2,3]; no BVD virus was detected (data not shown). Furthermore, pedigree analysis of affected calves revealed common ancestry on both maternal and paternal sides of the pedigree, thus implicating a genetic basis of disease.

**Figure 2:**
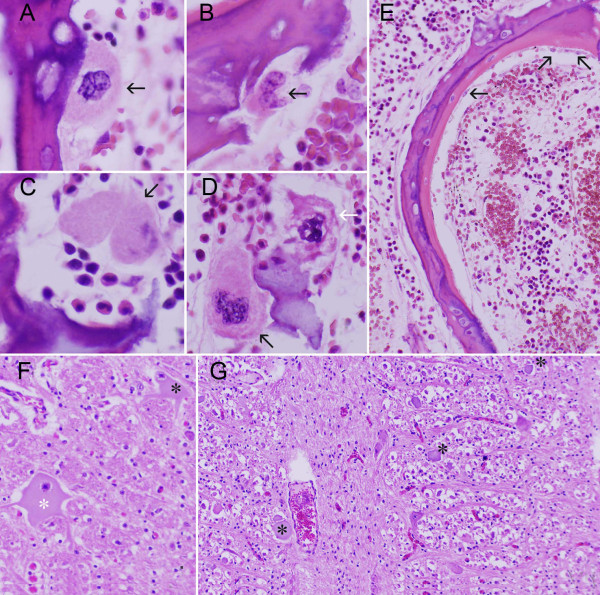
**Histopathology of bone and brain in late-term aborted Red Angus calves. A-D (distal aspect, femur)**: Abnormal morphology of few remaining osteoclasts persisting in bone. **A**. Osteoclast with scant cytoplasm and no obvious Howship's lacuna. **B**. Osteoclast with scant, lightly vacuolated cytoplasm that partially occupies lacuna. **C**. Osteoclast with nuclear degeneration. Myeloid cells are interposed between osteoclast and lacuna. **D**. Two osteoclasts associated with fragment of cartilage. One (black arrow; bottom left) is relatively normal. The other (white arrow; top right) exhibits nuclear changes consistent with apoptosis. **E (mid-section, rib)**. Scant, widely separated osteoblasts (arrowhead) associated with osteoid. **F-G (brain, medulla oblongata at obex). F**. Chromatolysis of large neurons (asterisks). **G**. Widespread axonal spheroids in reticular formation (asterisks).

Five affected calves were further subjected to histological examination. A marked reduction in the number of osteoclasts and osteoblasts in affected bone were consistently observed. Where present, osteoclasts were small, irregularly shaped and exhibited pale cytoplasm (Figure 2A-D). Howship's lacunae appeared shallow and in many cases osteoclasts were either absent or displaced from the resorptive surface. Additionally, cellular changes suggestive of osteoclast apoptosis were occasionally encountered (Figure 2D). Similar to the osteoclasts, osteoblasts were also markedly diminished in number and, where present, were small and flat with pale cytoplasm (Figure 2E). Histological examination of brain revealed consistent changes including widespread neuronal chromatolysis in cranial nerve nuclei of medulla oblongata, axonal spheroid formation in reticular formation, and perivascular mineralization in subependymal neuropil ventral to lateral ventricles (Figure 2F-G).

### Mapping of the osteopetrosis disease locus

To identify the defective gene associated with osteopetrosis, we obtained DNA from the first seven affected Red Angus calves acquired, their carrier parents and nine normal Red Angus controls. We initially examined the bovine orthologs of genes implicated in human disease for molecular variation associated with the osteopetrosis phenotype. The coding regions of the bovine *TNFSF11, TNFRSF11A, ITGB3, TCIRG1, CA2, CLCN7, OSTM1, CTSK *and *PLEKHM1 *genes were amplified and resequenced from the DNA of normal, carrier and affected animals (data not shown). No obvious causative variation was detected, thus warranting further investigation by whole-genome association analysis.

**Figure 3:**
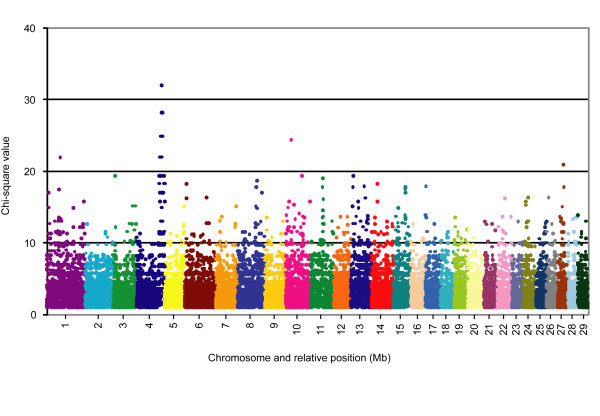
**Genome-wide case/control association analysis for the osteopetrosis disease locus**. Chromosome and relative position (Mb) are plotted on the x-axis, and the chi-square value calculated from a basic allelic test (1 df) is plotted on the y-axis. Any chi-square values less than 1.0 were not included. As shown, the most significant SNP associations are localized to the telomeric end of BTA4.

We then used the Illumina BovineSNP50 BeadChip array [16] to genotype each individual for >54,000 evenly-distributed SNP markers and subsequently performed genome-wide association and homozygosity analyses to determine the most likely chromosomal position of the disease locus. The basic case/control association analysis option of PLINK [17] was used to compare allele frequencies between the affected and normal animals. The most significant SNP associations were localized to the telomeric end of bovine chromosome 4 (BTA4; Figure 3). As this genomic location did not coincide with the locations of any of the original candidate genes, those genes were not examined further. Forty-two of the 100 SNPs with the highest significance of association were located between 108.37 Mb and 123.97 Mb on BTA4; the remaining 58 SNPs were distributed among sixteen other chromosomes, with a range of 1 to 8 SNPs per chromosome. Marker BTB-01 162852, located at 115.70 Mb, yielded the highest observed chi-square value of 32.00, while a group of five markers, located between 115.72 Mb and 119.72 Mb, showed the next highest chi-square value of 28.21 (Table 1). An allele frequency of 1 was observed among the affected animals, indicating that all affected animals were homozygous for a shared haplotype of these six markers that was less commonly observed among control individuals. The homozygosity mapping option of PLINK was also used to detect runs of homozygous SNPs from each individual, group individuals with overlapping regions of homozygosity and determine a consensus segment of matching alleles. The results of this analysis were consistent with the association analysis, revealing an extended region of homozygosity at the telomeric end of BTA4 that was unique to the group of seven affected calves. The combined group of overlapping segments spanned the chromosomal region between 90.65 Mb and 124.13 Mb, whereas the consensus segment included markers between 115.67 Mb and 119.32 Mb. Manual inspection of the overlapping segments revealed that the consensus region is actually bounded by heterozygous SNP genotypes at markers BTB-01163185 and ARS-BFGL-NGS-73498, defining a ~3.8-Mb interval from 115.61 Mb to 119.42 Mb (Additional file 1 Table S1). The nearest heterozygous genotypes on each side of this interval were located at 112.73 Mb and 119.76 Mb.

**Table 1:** Most significant SNP associations detected by case/control analysis.

Marker name	Chr	Marker position (bp)	MAF (cases)	MAF (controls)	Chi-square value	P-value
BTB-01162852	4	115,699,819	1	0	32.00	1.54E-08
ARS-BFGL-NGS-24470	4	115,725,375	1	0.05556	28.21	1.09E-07
ARS-BFGL-NGS-43056	4	116,685,970	1	0.05556	28.21	1.09E-07
ARS-BFGL-NGS-80140	4	117,048,104	1	0.05556	28.21	1.09E-07
ARS-BFGL-NGS-108296	4	119,189,609	1	0.05556	28.21	1.09E-07
ARS-BFGL-NGS-95252	4	119,272,013	1	0.05556	28.21	1.09E-07

"Chr" indicates the *Bos taurus *chromosome (BTA) number. "MAF" denotes the minor allele frequency, and is individually reported for case (affected) individuals and control (normal) individuals. The chi-square is the result of a basic allele test, with one degree of freedom (df). P-value is the asymptotic p-value for the basic allele test.

**Figure 4:**
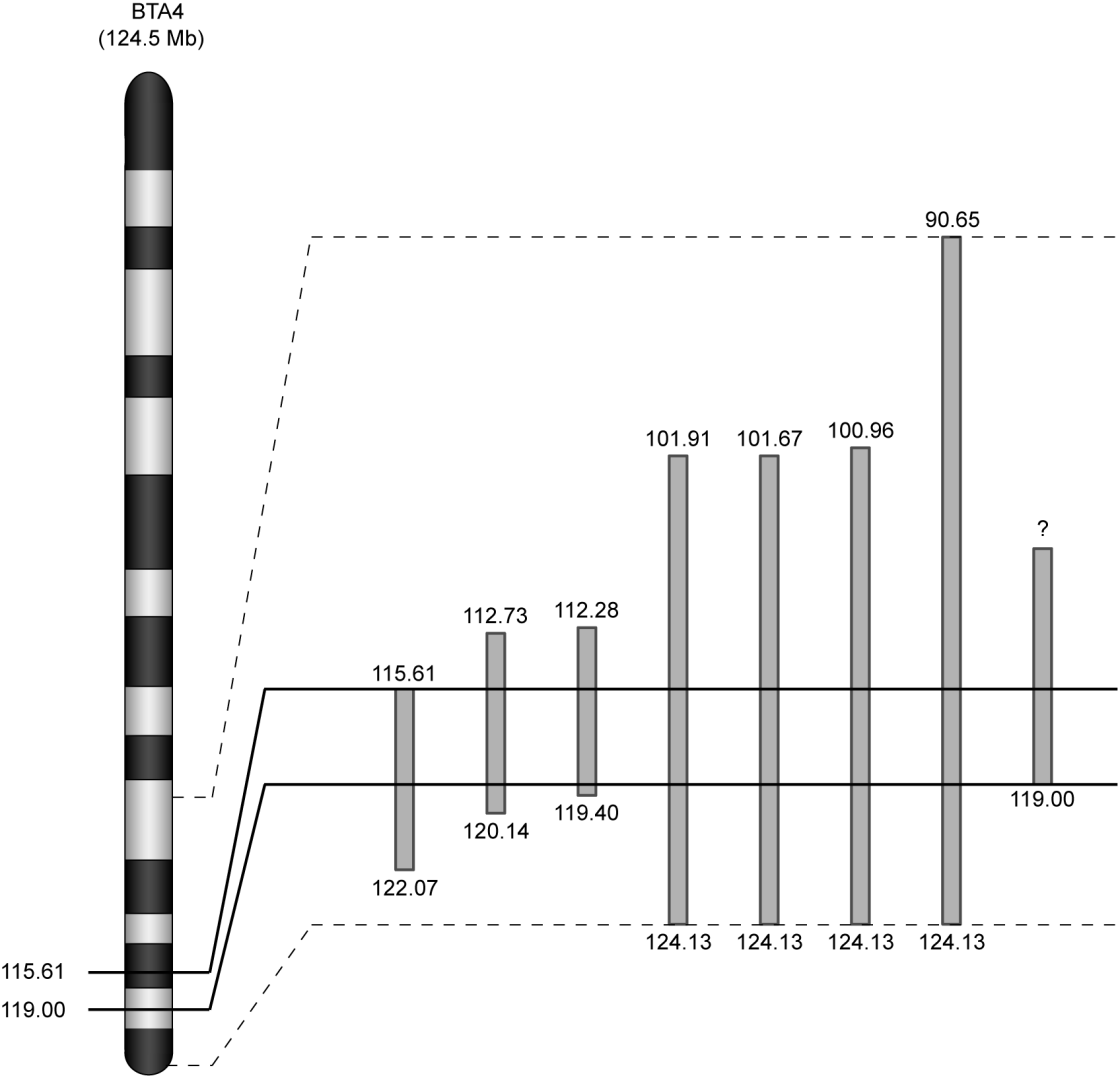
**Combined homozygosity analysis**. An ideogram of bovine chromosome 4 (BTA4; adapted from http://www.marc.usda.gov/genome/htmls/LinkageMap.jsp?Species=bos&Chromosome=4) is displayed alongside an enlargement of the extended regions of homozygosity detected in each of eight affected calves. Individual homozygous segments are depicted as gray rectangles, and the chromosomal positions (in Mb) of each bounding marker are indicated at the top and bottom of each segment. The homozygous segment on the far right represents one animal that was only genotyped for microsatellite markers between 107.14 Mb and 123.48 Mb; therefore, the extent of homozygosity proximal to 107.14 Mb is unknown, as indicated by the question mark. The overall region of homozygosity, from 90.65 Mb to 124.13 Mb, is bounded by dashed lines. The consensus region of homozygosity shared by all affected calves, from 115.61 Mb to 119.00 Mb, is bounded by solid black lines. Thus, the osteopetrosis disease locus has been mapped to this 3.4-Mb region. The overall figure has been adapted from [43].

Based on a conservative interpretation of the association and homozygosity analyses, we further examined the BTA4 chromosomal region from 107.14 Mb to 123.48 Mb through the use of novel microsatellite markers developed from the bovine draft genome sequence (NCBI Btau_4.0). All seven affected animals and their parents were genotyped for 46 microsatellite markers with an average spacing of 0.36 Mb (Additional file 2 Table S2). In agreement with the SNP data, haplotype analysis revealed recombination events at 112.55 Mb and 119.40 Mb. Subsequent genotyping of an additional osteopetrosis calf that was born while the study was underway identified a recombination event at 119.00 Mb, providing slight refinement of the distal end of the interval. A combined analysis of the microsatellite and SNP marker data, highlighting the refined ~3.4-Mb interval, is shown in Figure 4. Genotype data for the overall region of homozygosity, including the consensus segment of 87 informative markers, are provided in Additional file 1 Table S1.

### Candidate gene selection and analysis

The most recent bovine genome assembly (NCBI Btau_4.0) indicates that the ~3.4-Mb chromosomal segment of interest contains 66 genes. Among these, we identified two strong functional candidate genes, *ATP6V0E2 *and *SLC4A2*, at 117.02 Mb and 117.92 Mb, respectively. *ATP6V0E2 *("ATPase, H+ transporting, V0 subunit e2") encodes the e2 isoform of an essential subunit of a vacuolar-ATPase proton pump [18]. As mentioned previously, this type of pump is used during the acidification process of bone resorption, and mutations in the a3 subunit of the osteoclast v-ATPase are associated with osteopetrosis in humans and mice [19-21]. It has also been shown that inactivation of the d2 subunit results in mild osteopetrosis in mice [22]. Although the exact configuration of the osteoclast v-ATPase has not been determined, e2 transcripts have been detected in osteoclasts, suggesting possible involvement in osteoclast function [23]. The other, more compelling candidate gene, *SLC4A2 *("solute carrier family 4, anion exchanger, member 2"), encodes the anion exchanger protein located in the basolateral membrane of osteoclasts that is responsible for clearing excess carbonate ions and replenishing the supply of chloride ions during lacunar acidification. Three reports published during the course of this study have indicated that, in mice, SLC4A2 is required for both the differentiation and bone-resorbing function of osteoclasts, and importantly, mice lacking this protein exhibit severe osteopetrosis [24-26].

In an effort to identify molecular variation associated with osteopetrosis, we amplified and resequenced each candidate gene from DNA samples from normal, carrier and affected individuals, and subsequently compared the sequences of affected calves with the others. Although a number of SNPs were detected within *ATP6V0E2*, none appeared to be causative. One SNP was identified within coding sequence; however, this nucleotide substitution was not detected in the affected animals. Four SNP alleles unique to the affected calves were identified within the 3' untranslated region (UTR); however, the biological significance of this variation was not obvious. Efforts to resequence *SLC4A2 *revealed the potential for both an unannotated first exon and a large deletion mutation in the affected calves. While searching the NCBI database of Expressed Sequence Tags (dbEST; http://www.ncbi.nlm.nih.gov/dbEST/) for ESTs with sequence similarity to the human *SLC4A2 *transcript (GenBank accession number NM_003040), one EST (GenBank accession number DV927173) sequence contained a transcriptional start site located almost 2.5 kb upstream of the first predicted exon of the bovine *SLC4A2 *gene (GenBank accession XM_586817). Comparison of the bovine genomic and EST sequences suggested that the true *SLC4A2 *gene sequence contains an untranslated first exon and a partially translated second exon. While attempting to amplify fragments encompassing the 5' end of the *SLC4A2 *coding sequence (i.e., the start codon within the newly-annotated exon 2), allele-specific amplification was observed; no PCR product was generated from the affected calf templates. Subsequent use of redesigned primers for several fragments in the region also failed to yield product from the affected calf samples, warranting further examination of the upstream sequence. Downstream of the allele-specific amplicon, amplification was observed equally among all animal samples for the remainder of the *SLC4A2 *gene sequence. No seemingly causative variation was observed in these sequences; only one synonymous nucleotide substitution specific to the affected calves was observed in exon 14. These results suggest that a deletion of the 5' portion of SLC4A2 may be responsible for the osteopetrosis phenotype.

**Figure 5:**
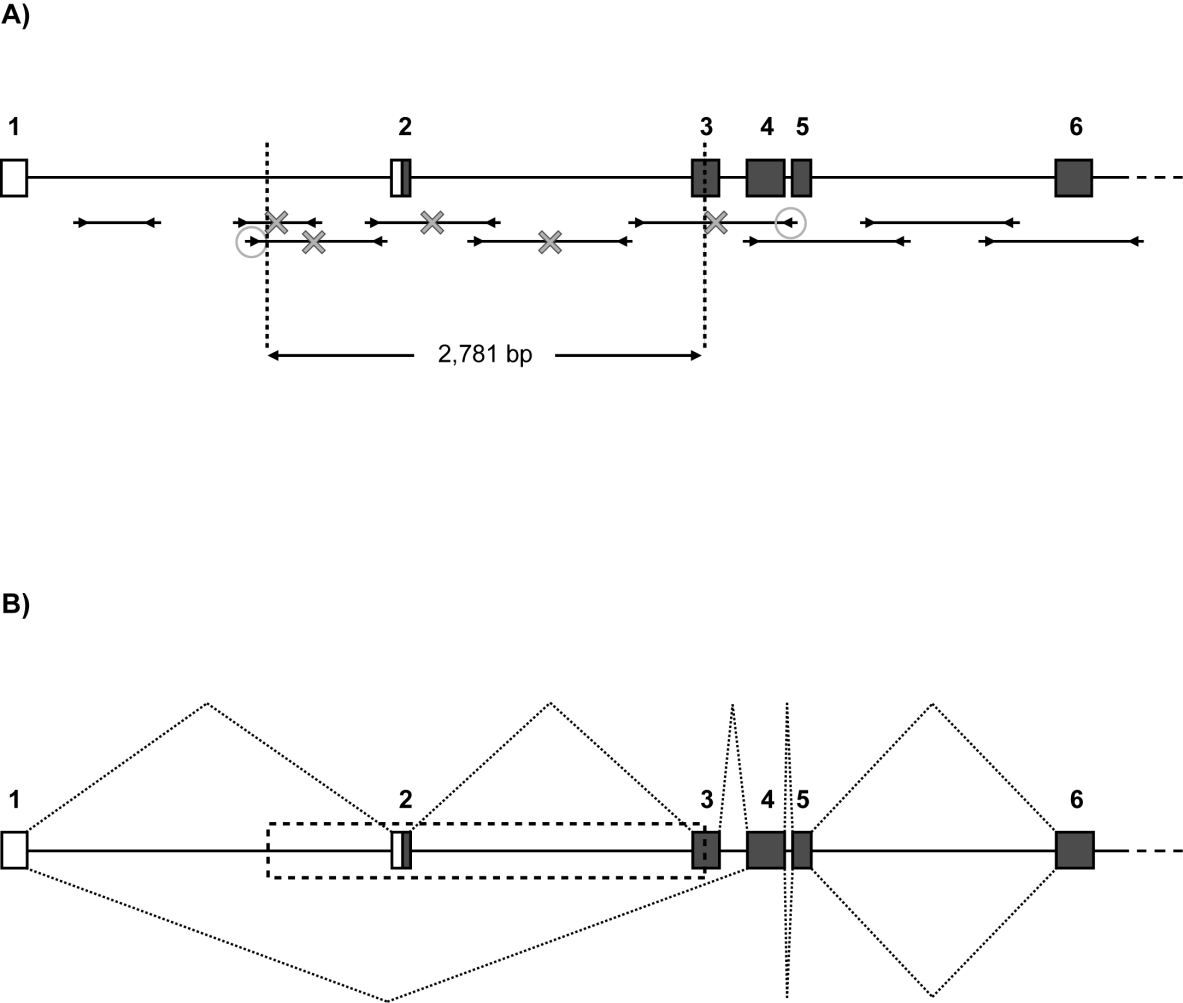
**Characterization of the *SLC4A2 *deletion mutation**. Each figure represents the first six of twenty-three *SLC4A2 *exons in the context of the genomic sequence. Each exon is displayed as a box, with the corresponding exon number indicated above. White coloring represents 5' untranslated region (UTR), whereas gray coloring represents coding sequences; therefore, the black line within exon 2 represents the start codon. Figure 5A depicts the determination of the deletion breakpoint. PCR amplicons designed to characterize the region are shown as fragments below the gene, and arrowheads represent primers. Fragments marked with an X failed to amplify from affected calf samples. The circled primers were eventually used to amplify across the breakpoint in affected calves. Direct sequencing of the resulting product revealed the breakpoint locations indicated by dashed vertical lines. Comparison with the normal *SLC4A2 *genomic sequence determined a deletion size of 2781 bp, as indicated. Figure 5B depicts the effect of the deletion mutation on splicing of *SLC4A2 *transcripts. The dashed box represents the genomic sequence missing from affected calf samples. The dotted lines above the gene reflect splicing of normal transcripts; splicing proceeds from exon 1 to 2 to 3 to 4, and so on. The dotted lines below the gene reflect splicing of mutant transcripts; due to deletion of exon 2 and the splice acceptor for exon 3, splicing proceeds from exon 1 to 4, and so on. Both transcript types were produced by a confirmed carrier cow, as detected by 5' RACE.

To characterize the breakpoint of the suspected deletion, primers were designed to amplify a series of evenly-spaced, short fragments upstream of the allele-specific amplicon (Figure 5A). Amplification patterns were then compared between normal and affected individuals. Upon observing equal amplification (i.e., from all animals) of fragments located on both sides of the allele-specific amplicon, different combinations of primer pairs, consisting of a forward primer from the upstream region and a reverse primer from the downstream region, were used to amplify across the breakpoint. Using one such primer pair, a product of ~700 bp was amplified from affected individuals; direct sequencing and examination of this product revealed a ~2,781 bp deletion (Figure 5A; Additional file 3 Figure S1). The sequence missing from the affected calves encompasses nearly one-third of intron 1, all of exon 2, all of intron 2 and nearly half of exon 3 (based on the new annotation derived from EST sequence). Importantly, the exon 2 sequence contains the distal portion of the 5' UTR as well as the first 17 codons. Although the exon 3 sequence is only partially deleted, the removal of the splice acceptor for this exon should result in removal of the entire exon (57 codons) from any transcript produced from the mutated genomic sequence.

### SLC4A2 transcript analysis

To test the hypothesis that the biological consequence of the deletion mutation is the formation of a transcript lacking exons 2 and 3, we performed 5' RACE to examine the cDNA ends of any *SLC4A2 *transcripts present in a known osteopetrosis carrier. RNA used for this analysis was isolated from the monocyte fraction of blood, which is known to contain mononuclear osteoclast precursors [27]. As expected of a heterozygous individual, both normal and mutant transcripts were detected. Normal transcripts displayed sequential splicing of the newly-defined exon 1 to exons 2, 3 and 4, whereas mutant transcripts displayed direct splicing of exon 1 to exon 4 (Figure 5B). Although variable transcriptional start sites were observed among 5' RACE products, no other transcript types were evident.

### Population analysis

**Figure 6:**
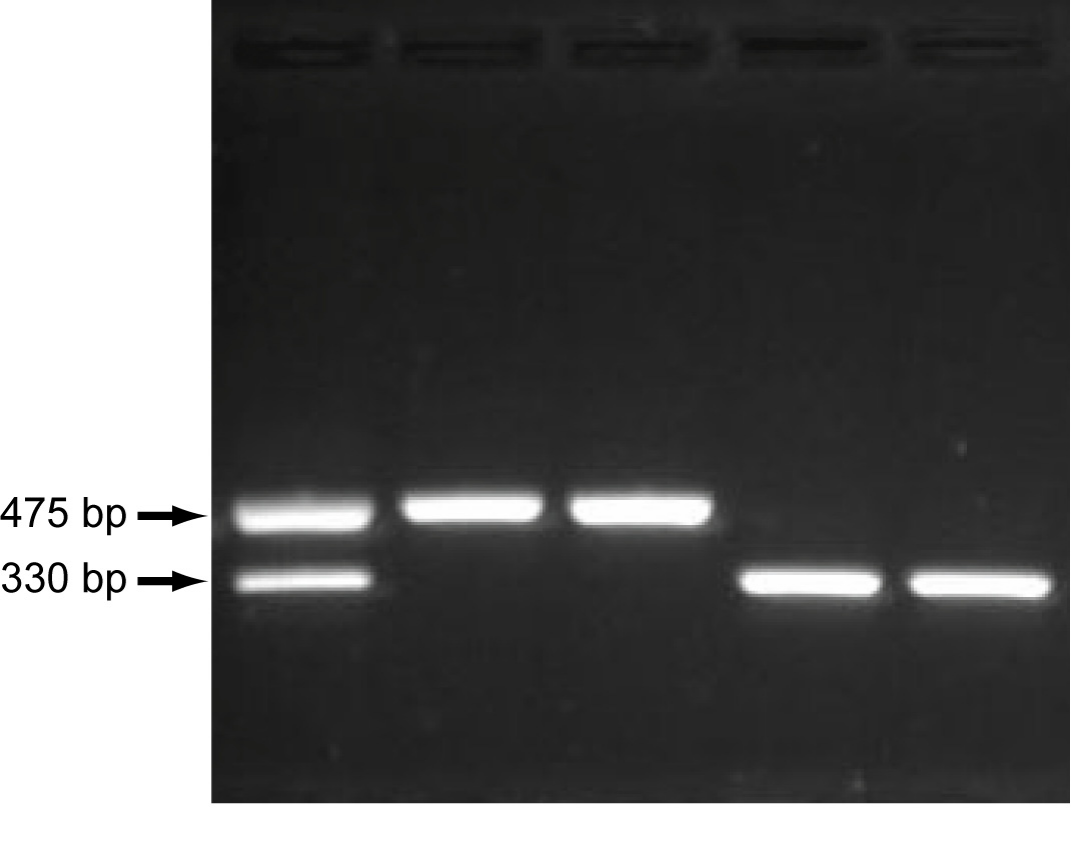
***SLC4A2 *deletion mutation genotyping assay**. Representative PCR amplicons for each deletion mutation genotype, separated by agarose gel electrophoresis, are shown. From left to right, the first lane represents a known osteopetrosis carrier individual, the second and third lanes represent normal individuals and the fourth and fifth lanes represent osteopetrosis-affected calves. As shown, both normal (475 bp) and mutant (330 bp) alleles are amplified from a heterozygous individual, whereas only the appropriate allele is amplified from homozygous normal and mutant individuals.

To further investigate or validate the association of the observed deletion mutation with the osteopetrosis phenotype, we designed an assay to genotype cattle for the mutation. A simple PCR-based assay was developed, based on the differential amplification of the normal and mutant alleles using a trio of primers (Figure 6; Additional file 3 Figure S1). A forward primer, used in combination with a reverse primer located in the deleted sequence, yields product only from the normal allele because the reverse priming site is missing from the mutant allele. The same forward primer, used in combination with a reverse primer located across the breakpoint, yields product only from the mutant allele because the normal product would be too long (~3 kb) to amplify under assay conditions. Using this assay, all seven of the original affected calves, plus all of six additional affected calves born during the course of the study were found to be homozygous for the mutation. Furthermore, any available parent samples were heterozygous for the deletion, thus demonstrating complete concordance between the deletion genotype and the disease phenotype.

In addition to analysis of animals with known genotype status, we genotyped 451 phenotypically normal Red Angus cattle for the mutation. Although more heterozygous individuals were identified, no animals were found to be homozygous for the mutation. Included in this analysis were 265 sires representing the diversity within the Red Angus breed; 25 of these 265 were identified as carrier individuals, corresponding to an allele frequency of 0.047. Since the Red Angus breed was developed from (and continues to incorporate germplasm from) Black Angus, we also tested 578 Black Angus sires but did not detect the mutation among these animals.

## Discussion

In this study, we have used the latest bovine SNP chip technology and association analysis to map the osteopetrosis disease locus to a ~3.4-Mb interval at the telomeric end of bovine chromosome 4. Both a case/control association analysis and a homozygosity analysis implicated essentially the same chromosomal segment on BTA4, centered at the 117-118 Mb location. Molecular examination of the *SLC4A2 *functional candidate gene at this position not only resulted in a new annotation of this gene, but also revealed a large deletion mutation within this genomic sequence. By analyzing *SLC4A2 *transcripts present in a known osteopetrosis carrier, we have confirmed the presence of a previously unannotated first exon identified in dbEST, and defined the biological relevance of the *SLC4A2 *deletion mutation. We have further demonstrated that this mutation is concordant with the osteopetrosis phenotype. Thus, we believe we have identified the causative mutation for osteopetrosis in Red Angus cattle.

At the level of genomic DNA, the osteopetrosis mutation results in the deletion of 2781 bp of the *SLC4A2 *gene sequence, and thus the removal of roughly one-third of intron 1, the entire sequences of exon 2 and intron 2, and nearly half of exon 3. This has been confirmed by the successful amplification and sequencing of the genomic fragment spanning the deletion breakpoint junction in affected individuals, followed by comparison of the mutant sequence with the normal *SLC4A2 *sequence. Inspection of the genomic sequence encompassing the deletion mutation did not reveal an obvious mechanism for spontaneous chromosomal breakage at this location. Deletions of this type can often be attributed to genomic instability; typically this instability is associated with the formation of alternative, non-B DNA secondary structures [28]. As a general rule, DNA sequences that form such secondary structures are repetitive [28]; yet, analysis of the *SLC4A2 *sequence with RepeatMasker http://www.repeatmasker.org did not yield masked sequences within the immediate vicinity of either chromosomal break (Additional file 3 Figure S1). The nearest repetitive sequences are at least 550 bp from either side of each breakage point, and only a few short (30-70 bp) repeats were detected within the deleted sequence. Thus, the cause of the deletion mutation is unclear.

At the level of transcription, the deletion mutation results in the production of mutant transcripts lacking exons 2 and 3. This was expected, given the deletion of exon 2 as well as the splice acceptor for exon 3, and has been confirmed by analysis of 5' RACE products from a known osteopetrosis carrier cow. Despite evidence of multiple *SLC4A2 *transcripts (referred to as types "a", "b" and "c") in other species, only normal and mutant type "a" transcripts were detected by 5' RACE. The ~4.4-kb type "a" transcript is the longest known *SLC4A2 *isoform, and has been detected in the human, mouse and rat [29-32]. Unlike the other transcript types, this isoform is ubiquitously expressed, and has been shown to be upregulated in osteoclasts [26]. It consists of exons 1 through 23, and the start codon is located within exon 2. Slightly shorter, ~4.2-kb "b" isoforms have also been described in the human, mouse and rat [29-32]. Both "b_1_" (human, mouse and rat) and "b_2_" (human and mouse) isoforms initiate transcription from alternative first exons, containing alternative start codons, that are located in intron 2. Expression of these transcripts appears to be predominant in the stomach, but also at reduced levels in other tissues; in humans, the relative abundance of these transcripts appears to be approximately 10% of that of the "a" isoform [31]. Therefore it is possible that no type "b" transcripts were detected in this study as a result of either a lack of expression in osteoclasts or expression at a level insufficient to be observed among the clones picked. If type "b" isoforms are present in bovine osteoclasts, it is expected that the deletion mutation would result in the removal of both alternative first exons as well as exon 3. Finally, a third, ~3.8-kb "c" type isoform has been detected in both mice and rats [29,32]. Similar to the "b" isoforms, "c_1_" (mouse and rat) and "c_2_" (mouse) isoforms initiate from alternative first exons; these exons are located in intron 5, and splice to exon 6, which contains the start codon. The "c_1_" isoform has been described as stomach-specific, whereas the "c_2_" isoform can be detected at low levels in other tissues [29]. In this case, the lack of observation of a "c" transcript type in cattle, and likewise in human, is likely due to the fact that the start codon utilized in mouse and rat sequences is not conserved. Both the human and bovine sequences contain a methionine codon further downstream within exon 6, yet, based on a lack of detection in either species, this does not appear to be a translational start. Although the nucleotides surrounding this codon may represent an adequate Kozak sequence, they do not comprise a strong initiator sequence [33].

At the level of translation, then, it is expected that the deletion mutation would result in a complete lack of bovine SLC4A2 protein expression. Although this has not been confirmed in this study, much can be inferred by comparison with recent studies of SLC4A2 knockout (Ae2-/-) mice. Mice deficient in all *SLC4A2 *isoforms, as a result of targeted replacement of exons 14-17 with A the neomycin resistance gene, fail to resorb bone and develop severe osteopetrosis [25,26]. The gross and histological features observed in Ae2-/- mice are similar to those observed in osteopetrosis-affected calves; Ae2-/- mice exhibit improper bone marrow cavity formation [24-26], growth retardation[24-26,34], early lethality (typically by age of weaning) [26,34], abnormal mandibles [25] and impaired tooth development [25,34]. In both SLC4A2-deficient mice and cattle, abnormal osteoclast morphology is apparent; however, histological differences are observed. In these mice, osteoclasts are enlarged with abundant cytoplasm [25,26], whereas the cattle osteoclasts examined here appeared small with a reduced volume of cytoplasm. Additionally, Ae2-/- knockout mice exhibit similar numbers of osteoclasts in bone when compared to wild-type mice, whereas we noted a marked reduction in the number of osteoclasts in the affected calf samples. This finding is consistent with previous reports of osteopetrosis in cattle [6,9,10].

Recent studies in mice have shown that the anion exchanger SLC4A2 is necessary for proper osteoclast function [24-26]. This finding suggests that, during normal bone development and remodeling, SLC4A2 is the osteoclast anion exchanger required to exchange bicarbonate ions produced by carbonic anhydrase II for chloride ions that are then transported across the ruffled border membrane, along with protons, to acidify the resorption lacuna and demineralize bone. It is expected, then, that a lack of SLC4A2 protein expression would prevent bone resorption due to the resulting lack of acidification. Indeed, studies in mice have shown that *SLC4A2*-deficient osteoclasts are unable to form an acidified vacuole [26], and in this study, the observed resorption lacunae are particularly shallow. However, unlike human osteoclasts harboring mutations in either carbonic anhydrase II [35] or the v-ATPase proton pump [19], which also mediate the acidification activity of osteoclasts, *SLC4A2*-deficient mouse osteoclasts appear morphologically abnormal and lack a ruffled border membrane [24-26]; thus, a bone resorption defect due to a lack of SLC4A2 may be more severe. Additional functional complications may result from the improper alkalinization of the osteoclast cytoplasm. It has been suggested that the expression of other proteins involved in bone resorption may be affected by a loss of SLC4A2, and indeed, abnormal expression of β3 integrin has been observed in *SLC4A2*-deficient mouse osteoclasts [26]. As β3 integrin is involved in the cytoskeletal organization step of the bone resorption process, this may explain the defect in ruffled border membrane formation. It has also been proposed that SLC4A2 may have multiple functions in the osteoclast, as another member of this protein family, SLC4A1, can function as a cytoskeletal anchor protein [26]. Thus, loss of SLC4A2 may affect the bone-resorbing function of osteoclasts through a number of different mechanisms; however, the effect of the cattle *SLC4A2 *deletion mutation on acidification activity is currently unclear.

Studies in mice have also shown that loss of SLC4A2 may affect the viability of osteoclasts. Although comparison of normal and Ae2-/- mice reveals similar numbers of osteoclasts, nearly four times as many *SLC4A2*-deficient osteoclasts show signs of apoptosis [26]; this has been attributed to cytoplasmic alkalinization. This finding is consistent with results reported here, and likely explains the marked reduction in osteoclasts observed in affected calves at the time of abortion. Histological examination suggests that, in cattle homozygous for the *SLC4A2 *deletion mutation, osteoclasts form but die prematurely by apoptosis. Several Howship's lacunae lacking associated osteoclasts were detected in the affected calf samples, suggesting that osteoclasts were once present, yet few osteoclasts were observed and cytologic features of apoptosis were evident among them. Unexpectedly, histological examination also revealed reduced numbers of osteoblasts in calf samples. Based on the increased bone density observed in these animals, osteoblasts were clearly present during fetal development. Thus, loss of SLC4A2 may also affect this cell type. Indeed, studies have reported intercellular communication among bone cells which regulates the balance between bone formation and resorption [reviewed in 36]. Interestingly, a pH-dependent model of osteoclast-osteoblast communication has been proposed in which SLC4A2 plays a key role [36,37]. In this model, extracellular alkalinization resulting from SLC4A2-mediated bicarbonate ion secretion activates osteoblast enzymes that degrade inhibitors of bone formation. Perhaps, then, a lack of osteoblast activation, due to the loss of SLC4A2 and apoptosis of osteoclasts, leads to subsequent death of osteoblast cells. Temporal differences in the death of these cell types could account for the overgrowth of bones observed in the affected calves.

It is interesting that no mutations in human *SLC4A2 *have been described to date in the context of osteopetrosis or any other hereditary disease. Approximately 30% of human patients with osteopetrosis have unrecognized molecular defects [15,38]; thus, it may be worthwhile to examine these orphan human osteopetrotic syndromes for mutations in *SLC4A2*. However, it has also been suggested that such human *SLC4A2 *mutations may result in embryonic lethality in humans due to ubiquitous expression of this protein, and as a result, will likely remain undetected [25]. For reasons unknown, mice and cattle lacking *SLC4A2 *exhibit differences in age at lethality; *SLC4A2*-deficient mice tend to die as weanlings, whereas cattle are aborted in late gestation. The abortion of affected calves is likely due to severe changes in brain, specifically compression of brainstem, herniation of cerebellum through foramen magnum, degeneration of caudal cranial nerve neurons, axonal spheroid formation, and mineralization of neuropil at the floor of the lateral ventricles. All of these features appear to be secondary to compression by the thickened osteopetrotic cranial vault. They have not been described, to our knowledge, as a congenital or antenatal defect in severe forms of inherited osteopetrosis in human patients.

We have demonstrated that the deletion mutation genotype is fully associated with the osteopetrosis phenotype in Red Angus cattle. Through the development and use of simple, PCR-based assay to detect the deletion mutation, we accurately identified 100% of affected calves and their carrier parents as such based on genotype. Due to this accuracy, the genotyping assay can now be used as a molecular diagnostic tool for the identification of other osteopetrosis carriers within the Red Angus population. In this study, population analysis identified 25 carriers among 265 AI sires; this allele frequency suggests that the frequency of the mutation in the Red Angus population may be as high as 4.7%. As a caveat, however, this estimation includes a group of animals submitted by one breeder in which nearly 30% were identified as carriers. Taking this into consideration, a more realistic estimate is likely to be on the order of 1.5-2%. Results of all tested AI sires have been reported to the Red Angus Association of America for dissemination to breeders. The accessibility of this information, as well as any future carrier information generated through the use of the diagnostic test, will allow cattle producers to make educated decisions for herd management that not only facilitate disease prevention, but also enable retention of valuable germplasm.

While assessing the Red Angus population, we identified a carrier bull that was born in 1964. This finding is consistent with the emergence of the disorder; however, as no ancestors of this bull were available for testing, the identity of the proband remains unknown. It is interesting to note that this oldest known carrier bull is a descendant of Black Angus grandparents, yet the deletion mutation could not be detected among 578 Black Angus individuals tested. This suggests that, if the same deletion mutation causes osteopetrosis in both Black and Red Angus cattle, the frequency of the mutated allele among Black Angus cattle is less than 0.001. Alternatively, it is possible that at least one other bovine mutation causing osteopetrosis exists. Any additional mutations that may cause this disorder in other breeds of cattle are currently unknown.

## Conclusions

We have identified a deletion mutation in the bovine *SLC4A2 *gene that is associated with osteopetrosis in Red Angus cattle. Discovery of such a mutation supplements current knowledge regarding the role of the SLC4A2 anion exchanger in the bone resorption process and furthers understanding of osteoclast biology. Additionally, the development of a DNA-based assay that can serve as a molecular diagnostic test for osteopetrosis carriers will facilitate the eradication of this disorder from the Red Angus population while minimizing loss of genetic merit. Furthermore, we have demonstrated the effectiveness of the bovine SNP chip technology for whole-genome association analyses and elucidation of causative genetic mutations, even when relatively few samples are available. Thus, this strategy can be applied toward the discovery of other disease loci in cattle and the goal of broadening the available set of genetic tools for management of recessive defects.

## Methods

### Animal samples

The diseased animals used were generally stillborn or died prior to discovery, or rarely were euthanized shortly after birth by the owners prior to being supplied to the authors for the study, so no animal care and use guidelines apply. Samples from unaffected animals were obtained from commercial semen companies or had blood collected by licensed veterinarians on the property and under the supervision of the owners of the animals. Thirteen Red Angus calves exhibiting the osteopetrosis phenotype were identified at the Wyoming State Veterinary Laboratory (WSVL), the Nebraska Veterinary Diagnostic Center and the private practice of Lou Scott, DVM. Detailed gross and histological examination was performed on five affected calves (four male and one female) following abortion in late gestation (215-230 days; normal gestation is 283 days). Tissues were fixed routinely in 10% neutral buffered formalin. Bone from axial and apendicular skeleton was decalcified. Five micrometer-thick sections of tissue were cut and stained with hematoxylin and eosin, and examined histologically.

DNA samples isolated from the first seven affected calves obtained were used for both SNP genotyping and fragment analysis of novel microsatellite markers. DNA from an additional affected calf born during the study was only genotyped by fragment analysis. DNA from an additional five affected calves, also born later in the study, were included in population analyses as well as validation of the deletion mutation assay. The seven affected calf samples used for whole-genome association analysis were offspring of two sires and seven dams; samples were collected from all nine parents. The two sires were full siblings; one bull sired three affected calves, and the other sired four. Five of these seven calves were produced from parent-offspring matings. Samples from an additional six affected calves and five putative carriers were also obtained. The nine control Red Angus samples used for SNP genotyping as well as some AI sire samples were obtained from the United States Meat Animal Research Center (USMARC) "2000 Bull Project". Other Angus and Red Angus AI sire samples used for population analyses were acquired from members of the National Association of Animal Breeders (NAAB) or from Gordon Jones (Western Kentucky University).

Samples were acquired in the form of blood, semen or tissue. DNA was typically isolated using commercially available kits (Zymo Research or QIAGEN) according to manufacturer instructions.

### Mapping of the osteopetrosis disease locus

Genomic DNA samples acquired from the first seven affected Red Angus calves, their carrier parents and nine normal Red Angus controls were genotyped using the Illumina BovineSNP50 BeadChip array [16]. Whole-genome association and homozygosity analyses were performed using PLINK [17]. The "--assoc" option was used to conduct the basic case/control association analysis; for this analysis, the "--cow" command was used to specify the *Bos taurus *species, and the "--maf 0.01" command was used to set a minor allele frequency inclusion threshold of 0.01. The "--homozyg" option was used to conduct the homozygosity analysis; the number of allowed heterozygous SNPs per window was set to zero ("--homozyg-window-het 0") and the number of allowed missing genotypes was set to twelve ("--homozyg-window-missing 12"). The grouping of segments of matching alleles was achieved using the "--homozyg-group" and "--homozyg-match 1" commands. The homozygosity analysis reported in Additional file 1 Table S1 was adapted from a verbose group file created using the "--homozyg-verbose" command.

Microsatellite data generated from fragment analysis was added to the verbose SNP genotype homozygosity file. The genotype file was manually inspected to identify heterozygous markers.

### Microsatellite marker development and fragment analysis

Novel microsatellite markers were identified using the online Simple Sequence Repeat Identification Tool (SSRIT; http://www.gramene.org/db/searches/ssrtool) to screen the bovine genome sequence (NCBI Btau_4.0) for repeats with a minimum length of ten per dimer. Fragment analysis genotyping was performed using a multiplex of primers, including one standard primer and one M13-tagged primer per marker primer pair, plus one fluorescently-labeled (PET™, NED™, VIC^® ^or 6-FAM™ dye; Applied Biosystems) M13 primer [39]. Markers were grouped into multiplex PCR reactions based on color and size combinations. PCR was performed in a 10-μl reaction volume containing ~30-50 ng of template DNA, 1× PCR buffer (containing 1.5 mM MgCl_2_; QIAGEN), 200 μM each dNTP (Fermentas), 0.25 μM each standard primer, 0.0125 μM each M13-tagged primer, 0.15-0.25 μM each fluorescently-labeled M13 primer and 0.25-0.35 U HotStar Taq DNA polymerase (QIAGEN). Typical PCR cycling parameters included an initial denaturation step of 95°C for 5 min followed by 35 cycles of 94°C for 1 min, 58°C for 1.5 min, and 72°C for 1.5 min, plus a final extension step of 72°C for 1 hr. Primer sequences and marker information are provided in Additional file 2 Table S2.

Multiplex PCR products were combined and purified using Promega Wizard^® ^SV96 binding plates as described [40]. GeneScan™ 500 LIZ^® ^size standard (Applied Biosystems) was added to 10-μl aliquots prior to loading on an ABI 3730 capillary sequencer. Automated allele-calling was performed using GeneMarker^® ^(SoftGenetics, LLC) software. Allele calls were checked manually and edited if necessary.

### Primer design and PCR

Primers used in this study were designed using Primer Designer 2 (Scientific and Educational Software). Standard PCR and cycle sequencing reactions were typically performed as previously described [41]. Primer sequences for *ATP6V0E2 *and *SLC4A2 *candidate gene sequencing are provided in Additional file 4 Table S3.

### Candidate gene analysis

Candidate gene fragments were amplified and re-sequenced from a select group of eight Red Angus samples of known or suspected genotype status; two samples represented normal animals, two samples represented affected calves and four samples represented putative carrier individuals. These sequences were then aligned and compared to the genomic sequence to identify polymorphisms. First, the genomic sequence of each candidate gene was masked of repetitive elements using RepeatMasker http://www.repeatmasker.org. Primers were then designed in non-repetitive sequence to amplify and sequence overlapping ~1-kb DNA fragments. PCR was performed, and products were treated using a 1:10 dilution of ExoSAP-IT^® ^(USB Corporation) as described [41]. Approximately 100-200 ng of each purified PCR product was then sequenced directly, using each primer used for amplification, as described [41]. Sequencing product was then purified by size exclusion with Sephadex™ G-50 Fine beads (Amersham Biosciences) prior to loading on an ABI 3730 capillary sequencer (Applied Biosystems). Sequences were analyzed and polymorphisms were detected using CodonCode Aligner http://www.codoncode.com/aligner/ or Phred/Phrap/Consed software [42]. The genomic sequence of each candidate gene was included as a reference. All sequence chromatograms were visually inspected to confirm detected polymorphisms.

### Transcript analysis

Total RNA was isolated from blood taken from a cow known to be heterozygous for the *SLC4A2 *deletion. Whole blood was first mixed with approximately six volumes of an erythrocyte lysis buffer (155 mM ammonium chloride, 10 mM potassium bicarbonate and 0.1 mM EDTA). The remaining leukocytes were then pelleted and resuspended in TRIzol^® ^Reagent (Invitrogen), and RNA was extracted according to the manufacturers' instructions. Following RNA purification using the RNeasy Mini Kit (QIAGEN), the 5' cDNA ends of bovine *SLC4A2 *were generated using the FirstChoice^® ^RNA Ligase Mediated Rapid Amplification of cDNA Ends (RLM-RACE) Kit (Ambion). Gene-specific primers were designed within exons 4, 5, 7 and 8 based on a hybrid sequence of an available bovine *SLC4A2 *EST (GenBank accession number DV927173) and the predicted coding sequence (GenBank accession number XM_586817). The EST sequence was used to obtain the 5' UTR sequences of exons 1 and 2 and the predicted sequence was used for the coding regions of exons 2-23. Following RNA modification and reverse transcription, the supplied 5'-RACE-Outer primer was used with exon 5 primer SLC4A2_5R_743C (5'-GTGGAGAGGGACTGGTGGTT-3'), exon 7 primer SLC4A2_7R_1060C (5'-CCCAGGAACATCCTCAAACC-3') or exon 8 primer SLC4A2_8R_1213C (5'-TTTGTCCAGCAGCAGCTCAT-3') to amplify primary 5' cDNA end products; these products were then used as template in secondary, nested PCR reactions using the supplied 5'-RACE-Inner primer with either exon 4 primer SLC4A2_4R_468C (5'-TGGATGTGATGGGAAGACTG-3'), exon 5 primer SLC4A2_5R_743C and exon 7 primer SLC4A2_7R_1060C, respectively. Nested PCR products were cloned using the TOPO^® ^TA cloning kit for sequencing (Invitrogen, pCR^®^4-TOPO^® ^cloning vector). Cloned cDNA end products were analyzed by restriction, and clones of differing insert size were bidirectionally sequenced as described [41]. Sequences were analyzed using CodonCode Aligner.

### Deletion mutation genotyping assay

A deletion mutation genotyping assay was developed based on the differential amplification of the normal and mutant alleles using a trio of primers. A forward primer (5'-GGGAAGGGAAGCACTAAGACT-3') was used in combination with a reverse primer located in the deleted sequence (5'-TGGAGAGACAGCAGCAGAGAT-3') to yield a 475 bp product representing the normal allele, and a second reverse primer located across the deletion breakpoint (5'-GGTGGATGTGATGGGAAGACT-3') to yield a 330 bp product representing the mutant allele (Figure 6; Additional file 3 Figure S1). PCR was typically performed in a 20-μl reaction volume containing 30-50 ng of template DNA, 1× PCR buffer (containing 1.5 mM MgCl_2_; QIAGEN), 200 μM each dNTP (Fermentas), 0.5 μM each primer, and 0.25 U HotStarTaq DNA polymerase (QIAGEN). PCR cycling parameters included an initial denaturation step of 95°C for 5 min followed by 31 cycles of 94°C for 45 s, 63°C for 45 s, and 72°C for 45 s, plus a final extension step of 72°C for 5 min. Products were separated on a 1.6% agarose, 0.5 × TBE gel with a 4 cm well-to-well distance (Figure 6).

## Authors' contributions

DJS, DO, and SLS shared responsibility for collection of samples, diagnosis, and histological examination. JEB, TPLS, and TSS constructed pedigrees, collected pedigree samples and control DNA samples. BMM, SNM, TPLS, and TSS performed sample processing and DNA extractions. SNM, TGM and TPLS screened for polymorphisms and performed genotype and diagnostic PCR assays. TSS performed Illumina beadchip assays used for homozygosity analyses by BMM, JRO and SNM. SNM identified the mutation and determined the deletion endpoints. JEB and TPLS were responsible for study design and supervision of the project. SNM drafted the manuscript with input from all the authors. All authors read and approved the final manuscript.

## Supplementary Material

Additional file 1 **Table S1: Combined homozygosity analysis**. Click here for file

Additional file 2 Table S2: Novel microsatellite marker primers. Click here for file

Additional file 3 **Figure S1: Red Angus Osteopetrosis Disease Locus Sequence Information**. Figure S1A displays the bovine SLC4A2 genomic sequence encompassing the deletion mutation associated with osteopetrosis in Red Angus cattle. Exon sequences are bracketed and highlighted with green bold text. Exons 1-4 are shown, and the start of exon 1 corresponds to the 5' transcriptional start of GenBank accession number DV927173. Splice donor (GT) and acceptor (AG) sites are highlighted in black bold text. The start codon within exon 2 is highlighted in red bold text. The 2781-bp deleted sequence is shaded gray, and each breakpoint is marked by a bold double slash (//). Sequences denoted with a double strikethrough were identified as repetitive by RepeatMasker. Figure S1B indicates the amplicon sequences generated from the PCR-based deletion mutation genotyping assay. Amplicon sizes in base pairs are noted. Primer sites (sense strand) are underlined. The deleted sequence is denoted by the triangle symbol (black triangle). Click here for file

Additional file 4 Table S3: Candidate gene primers. Click here for file
