# Supplementary material for: A deletion mutation in bovine SLC4A2 is associated with osteopetrosis in Red Angus cattle
Source: BMC Genomics. 2010 May 27;11:337. doi: 10.1186/1471-2164-11-337 (PMC2891616; doi:10.1186/1471-2164-11-337)
Supplement: Additional file 3 — Figure S1: Red Angus Osteopetrosis Disease Locus Sequence Information. Figure S1A displays the bovine SLC4A2 genomic sequence encompassing the deletion mutation associated with osteopetrosis in Red Angus cattle. Exon sequences are bracketed and highlighted with green bold text. Exons 1-4 are shown, and the start of exon 1 corresponds to the 5' transcriptional start of GenBank accession number DV927173. Splice donor (GT) and acceptor (AG) sites are highlighted in black bold text. The start codon within exon 2 is highlighted in red bold text. The 2781-bp deleted sequence is shaded gray, and each breakpoint is marked by a bold double slash (//). Sequences denoted with a double strikethrough were identified as repetitive by RepeatMasker. Figure S1B indicates the amplicon sequences generated from the PCR-based deletion mutation genotyping assay. Amplicon sizes in base pairs are noted. Primer sites (sense strand) are underlined. The deleted sequence is denoted by the triangle symbol (black triangle). [file 1471-2164-11-337-S3.DOC]

**Figure S1. Red Angus Osteopetrosis Disease Locus Sequence Information.**

**A)**

>*SLC4A2* 5’ genomic sequence

ACCTTACAGAGCCCCGGCCCACTGGAGTCGGCCAGGGTGTGGCCCTAGAGCTGGCAGGTTGGTTACCGCCCTCAACAGACAGAAGGACAGACAGAAGCTACACTTGAAGCGGCCTTGACCTCATCCCTTAAGGAGTTGATTATAGATTTATCTTGTGGCTTCTCTCTAATTGTCTCATGATTGCATTCTTGAGGGCTGTGAACTGGATGAAAACTGCCCTCAAACAGCACTCCTCGGCGCCGCCGGAACCGACGTAGGGGCTTAAACAGTTTGTTAATTAACTCGTTTATGTCTGAACGTTTTCTGCGTACACAATGCTGTCCTTCCCTCGGTGGCAGACTCTCAGAAGCGAAACATAGATTCGCTTATCTGGAAGAATTTATAATTTAAGAAAACAAGAAATAGATCTTTAAATGACAGGTGATGACTATGAGTAGGGTTCGAGAAAGGAGGTCATGGCAGGGAATCTCTCCGAGGCAGAAGTCCCCCGGTTCGGAAAGACTTAGCACAGAGCAAGGGGTATCCAACCCGCGCCGCCATCACCGCTCTTCGACCCCACCCCCGTCCTCCTGGCAAAGATAGGGGCTTTCTTGGAGGCACTGGGGACTACAAAGGGTGGAAGGCTGGGGCTGCCTGCGGGCGCTGAGAGATTCTTGACCCCCGATGCATTGATATTTCAATTTCCTCCGAATTGCCTAATAGCTTGGTGCCTCTTTTTTTTTTTTTTTCCCCCCTGCCTGGGATTCCTAGGATGAGGCGGGCTTCCTTTCCGCCCAGAAGGGCCAAATGAAGCTAGGAGACGGCGTCTCCAGGCACCCGAGCTCGTTGCGGGGAAGCGCTGGGCGGCCGCCTCCCGCCGCGCGGAGGGTCAAGCCAGGGGCGGCCTCCTTGGCTCTGGGATCCGGGCGCCGCGCAGTGCTTGGCCGCGCGCCTCCGCCCCCTGGGTCTCGGCCCCACTCCCCGCCTCCAGGCCCGCCGCGCCGGGAACGCGCACGGTGCGCCGGGGCGCGCGCACGTAGGGGGCTGGCCTGCCCGCGA**[CGCGGGGGAAAGTTGAGTTGGGAGAAGTTGGGAGCGGCGGGGGGCGCGCCCCGAGGTGGGCCCGGGGGAAGCCGTCGGGAGCGTGCGAGGGCTCCAGAGCCGGCGGGGAGAGAGACCCCCGAGACCCCTGGAGCGCCAGGTCCAGAGGAAGCGG]GT**AAGCTGGGAGGTGGCGCCGGGGGAGGGCTGGGGCCGCGGAGAGAGGAAGGGAGGGGGAAGGAAAGGGGGGAACTCGGCACTCGGAGCGGGGAGGGAGTGGAAGTGCAGGAAAGGGCGGAGACTGGGGCGCGGGGACGCGAGCTTTTGGGGGAGAAGGATGGGGAGGCAAGCGGAGTGATTGCGGGTCGGAAGAGGGGTCTAGAGTCCGCTTCTTCGAGTTCCCTAAAGCGAGGGTGCCGCGCCCCCTGCCACCGGGAGCGGGTACTCTCTGGCAGGCGCCGCCGGCCCTCCCGAACCCGGGCAGTACTCCAGACGACACCCTTCCCGTGCCACCCCCTACTCTAGCCTTAAGGCCCGTCCAGGGCGCCCCTGCCTAAGAGTTGCCCCGAGCCTCAGCCTCTGCCCCCCAATCTCGGGCTTGGGAGGCGGAGCCAGGAATGGGGGTGGCGGGTCAGGTCGGGACAAGGGGTCGGGCCGGAGAAGGAGCAGCTGGGTGGTGATGGGAAAGACGAGCCCTGGAGAGTGCGACCTAACTAACTCCTGGCGGGAGTGGGAGAGGGGGTGCTGGCTGGCCCGGGTGGGGAGCTCTGTGCTGGGTGTAGGGGCACGGCGCTGACCTGCACTCCAGGGTCCACTTTCTCCTCCTCCCTGAGCTCCCCACTCCCGCCAGCCCTTCGTCAGGAGAGCGGTGGAATGTGAGTCCACTTGGGGGGCGGACGGCGGTGGTGATTAATGCCCAAGTGGGGGTCCGGAGTGCTAATCCTCCGGGCCGGTCGGGGCTGGTCCCTGGAATGTGCCCCCACCCCGGATTATCGACACCGATGCTCTGGACAGAAGCAGCTTTGTGTTCTGGGGACAACGCGGTGGGGGTAGGGGGAGGTAGAGGGAGGAGGGCGGGGGAGGACGGAGGAGCACACCAAGAGCCCAGTGGGAAGTGGGAGCGGGGCACGGGCAGGTGCCAGGCACTCCCTCCCAGACCCAGTTGTCTTTTCCAGTGCCCGGCGCCCCGACAGCAGACTTGCCCTGGCGCAGAGCCTGCTAAGAGGGGAGCTGAAGAGAGCACTCAGCTGAGCTGGAATGCCCCGCTAGGGGCATGTGTGGCTCCAGGCTCCCCACCCTGGACCGACCTATATTGATTGACCCTACACTGACCTGTGTCACCCCTGAGTTGGCCCTAAATCCGGTAACCCTGACCACCACTCCGACACCACCTCTTGCCCGGGGAGCTCAGTTCTCTTGCCATCACAGAGCACCCCCTCCGCTAGGAATGGGTCCCCCCCAGTTTGCTGGTCAGCTTCCCTAACCGCAGCCCTTCTGCTGGCCTCCTTCCGCGCCCGGTGGCCTCCCCAGAAGCCCCTACAGTCACAGTCAGGAGGGCGTCAGCCCGGCCGCTCAGATTTTCCTGGCCGCGGGTTTGGGGAAGGGAAGCACTAAGACTGCCCCCAGGGCAGAACCTCGGGGTTTCTGTGCCCCCTCCT**//**CCCCGGGCGTCACGTGCCCCTCCCCCAGTATAAAGCGCTGCCCGGCCAGTGCCGCCCGCCCGGCCGCCCTCCCACTGCCAGAGTGGAGCTGGGCTCCTGCCCCTCCCTGGGAGCCTCCTCCTCCCCCACTTCGGGGCTGGGCTTTTTTTTTTTTTTCGGAGGAGGGGGGGCACAGGACAGCAGCCCCCTTTGCGGGTTGCTCTGGGCTCACTCCAGAGTTCTGTCTGCCTTCCTTCATCCCCCTTTGCCCCGGCCCAGCTCTGGGTCTCACCCCCTACCCCTGTACACCCGTCTCCGGTCTCAGCCCCCTCTGCCAGCCGTGACCAGCCCCCTCCGCTCTCACTGCACTTCTGGCTCGGCCTGTCCTCCCTCTTCCTCTATCTCCCCAAGCTGATCTCTGCTGCTGTCTCTCCAGGTCTGGGCCTGTCTCCTTCCTGCCCCCAACCCCAGGGGTCTCCCACCCTCTTCTCTCTCCCCCTGCCTCCGTTCAGTCCCTGGGCCTGCTGGGCCTCCATCCTCACTTCAGTTCTTTGTTCCTCTCTTTTCTTAACTTTCCTGTCTTAACTTTCTCTGCTCCCCGCTCCACCCTCCCAGAGTCAGGCGCCAAGGCCCTTGGGAGCCTGGGAGGATCGTGTGCAAGCAGGAGACCCCCCCACCCCCGCAGGGCCCTGCCCTCGCCTGGGCTGGTCTGGGCTGGGGGAGCTGTGTGATGTGGGGGTGCTCCCGCTGGCCTGGGGCCTCTCTCACCTGGTGATTGGTGCCCTCCCCC**AG[GTTATGCCGCCACCTCGTCTCCCTGAAAGGCGCAGCTACAGCGAGAAGGGCTAAGATTTGGCCATGAGCAGCGGCCCCCGGCGCCCCGCGTCGGGCGCGGATTCCTTCCGAACG]GT**GAGTATTGCTCACTGCTCCTGTGCACCACCATCGCTGGGGCTGTGGCTGAGGGTGTGTGAGTGAAGAGGCCACCACTGGGGCTTTGGCCAAGGGTGAGGGTGTGTGTGTGTGAGTGAGAGACCGCCACTGGGGTTCTGGGCGAGTGAGGGTCTGTGTCAAACGGGTGTGAGTGTGTGTGCGTGTGCGCGTGTGAAGAAGCCCGAGAGGGGAGCCTGGATGGACTTGGGAAGGGCATTGCCATGCCTCTCCTTGCACGGGCCACCGGGTCTGGGACAGGTACTGGTGGTGGCGGTCAGATGGCTTCCAGCCCTGGGGAGGGCCTTGCGGCTCTGTTCCCTGAAGCCCTGGGAGGTTGTTTATCCTCCTCAGCGGGAGAGGAGGAACTTTGGGAATCCTGGGTCTCTAACCGATGACAGCCCCCTCCCCCGCCCTGGGTCTTGGAGGCTGTGCCTGCCGGGCCTGCTGCAAGGGGCTTCTGGGCAGACCGCCCCGCCCCCCGGCCCCACGTGCCACCCAGGCCCGGCCCCTCCCGGCCCCCACGTGGCCGCCGCCCCACGTGTCCCCCTTCCAGTGTTCCTGGGAAGGGCAATGATTAACCCCAGCGCCGGCATCATCCGCGCCTCATTGGTCACTTGGGCCCACGGGGGCACGTGACTGGGGCGAGGTGCCGGCCAGTCCCCTCCCCCTCCGCCCCCAGTTCACCCCAGCCCGCAGCCATGGACTTCCTCCTGCGGCCTCAGGTGCGAGGGGCTCTCTGCCCTCCTCCCCCCACCCCGGGCTAGCCCCCTTCTGCTCGCTGGCTTCAGCCGCCCCGCTCTACCGCCTTCCTCCGGTGCTCTTTCTGGTGAGAGTGGGGCCCCGTGCCTGGGGGTAGTTGGCGGAGAAGCCGGGCTCGAGCGGCAGAGATGGGCTGACCAGGGTTGGGGCGGGGGGTGCAAGCTTCTGGTCCTGGCCTCTAGTATCCAGTGTGTCCCCCCTCCCTGCCCCACCCAGCGGCTCCTGACCCCACCTCCCAGGCTCTGCAAGGCCCGCCCTCCTTCCCAGCCCACCTCCCTCCGCTCTTTCTTGCTCGGGTACCCCTGCCCTTGGAGATCTTGATGTCCTTCAAGTCCAGAAGCTCTTGCTTGAGCAAATATTTGGTGGTGCCTGGAGCTGAGACCAGCCGGCAGGGGGTGGTGGTGGTTGGGGGGGCGCTGGACCAAAGCTGCAGAGTGGTGGCTGCCTGCACTGCTGGGCTGAGCTCTCCGAAGCGCCACCACGGTCCTGGGCTCCCGCAGAATGACTCAGGTGCGGGGGGGGGGGGGGGGGGTGGAGCGGGGCGGGGCAGGGCTGCAGCCCAGGGCGCTGGCTCCCTCCGGTGGGCTTCCAGTTGACTCAGACCGAGCCCGGTCGGTTGGTTAGCTCAGGCGCCGCACCCCCCCCCCCCCACCAGGAGTTACTTGGCTCAGTCGGACAGGTCAGGGTCGCATTCACGACCATCAGTGTCAGCCATGTGAACGGCCCAGGTCACACCTCCGACCTTGACCTCCGGGGTGGACACACGGGACTTCTGCAGAGGCCTCCAGGGCTGACGCTTCCCACCTGCCCTAGAAACCGACAGGAATGGGCCCAGCCAGGGTGCTGATGGCCCAGGACTCCGGTGGGGGGGCGGGGAAGGCTGCTGGGGGTAGGGTGCTGAGTCCCCGGCTTTGGAGTCGGGGCAGCTGGGGGAAAGGTACTGGATTTCTTCTGCTAGGCGGGCAGGGGGTGTGCTGGGACACCCCCCCCACCCCCACCCCCGCTCCCCGAGAGTCCTCACTGCCCCTTCTTCCTTGC**AG[CCAGAGCCTGAGAGCCTGGGCCCCGTGACTGCACCTGGCTTTGCAGCTGAGCAAGAGGAGGATGAACTCCACCGCA//CCCTGGGCGTGGAGCGGTTTGAGGAGATCCTTCAGGAGGCGGGGTCCCGAGGAGGGGAGGAGCTCGGCCGCAGCTACGGGGAGGAAGACTTTGAAT]GT**GAGGGGGTCGGGGGAGGCGAGTTGGGGGCTGACAGGGTGTTGCTCCGGGCTGGGGGCCTGGGGGCGGGGAGAGGCTGGGGGCCCGCCAGCTGGAGAGGGGGGAGCAGGGCCCATTGGCAGCCAGTGCACCTGTCCCTGC**AG[ACCATCGCCAGTCTTCCCATCACATCCACCACCCGCTGTCCACCCACCTGCCTCCTGACACCCGGCGCCGAAAGACACCCCAGGGCCCAGGGCGGAAGCCCCGCAGGCGCCCCGGAGCCTCCCCGACTGGGGAGACCCCCACCATCGAGGAGGGCGAGGAAGAGGAGGATGAGGCCAGTGAGGCCGAGGGGGCTCGGGCCCTCAGCCAGCCTTCGCCTGCCTCCACACCCTCTTCCGTGCAG]GT**GAGCTGGGCAGGGCTCCCCGGGGGTGGGAGCGCTATCTGCAGGATCTCTGGCCCAACCTCCCCTGCTCACACGGCTTCCTTGTTCTAGTTCTTCCTCCAGGAGGACGAAGGTGCCGACCGGAAGGCAGAAACCACCAGTCCCTCTCCACCTCCACCCCTGCCCCACCAGGAGGCAGCTCCCCGGGCCACCAAAGCAGCCCAGACTGGGTAAGGGTCCCCACACAGAGACCCCCCATGCCAGCATCGGTCAGGTGGGGTGAGGAGACAAAGCAGGAGCCTTGAGAGGCTCAGGCTGTGGTTCAAACCCAGTTATGCTGCTAGGACTTGACTCTGGACAAGAACTGACGTCTCTGAGTCCATTTCCCCGTCTGTAAACCAAGCGTAATAGGACTTTTCCCACAAGGTGGTAGTAGGTTTCAAGGGTGGTGTGAATGAAAGTTTTAAATATGGTCTAGGCGCCTCCTCCCACACCTGTCATCTCCCCCCCAGCCATGGCTTCAGGCGAGCAATGTCAAACACAGGTCAGAGGCTGGACAGGTGACCTCCTCACACCCCCGGGACCCCAAGGGGGTGAGAGGAATAGGAGGCCCCACTCTTGCTTTTGGGGAGTTGCCAGTCCACCCTGGCTGTGGTCTCGTGAAGTGGCAGTCAGCACAAGTTACTGCGCCAGACGACTGGGTTCAGGCGGAACATCGGTACCAACAGGTGATTGTGGAGTTTGGGGACATCAGCCAGGGAAGGCTTCCTGGAAGAAACCACTTTGAACGAGGTCTCAAGGTCACTACTATCGCCTCCCTGTTGTCCGGGTGCCAGGCAGTGTGAAGGGGTGGGGGGAAGTGAAGGGCAAATTGCCCCTCCGGGGCGGCACAGACTCGCTGGGGAGACAGGCCGTGTGGATGGAGCCAGGTCCACCTCAGCCGTCTGCGCGGGAGGCGCAGGCCTGCGGGCCAGCAGATGGCCAGGAGGCGGGGGAACCCAAGAGAGAACAGGGCGTCGTACAAGGGGTTCAGATTTGTGGGCAGGAGTGTGAACAGAACCTCGGGCACGGAAGGTGGGTGCAGGTGGAGAGAGCCTGGCTCAGGGGGCGCTGGTCACGGCGCGGGACAG

**B)**

>Deletion mutation genotyping assay normal allele amplicon, 475 bp

GGGAAGGGAAGCACTAAGACTGCCCCCAGGGCAGAACCTCGGGGTTTCTGTGCCCCCTCCTCCCCGGGCGTCACGTGCCCCTCCCCCAGTATAAAGCGCTGCCCGGCCAGTGCCGCCCGCCCGGCCGCCCTCCCACTGCCAGAGTGGAGCTGGGCTCCTGCCCCTCCCTGGGAGCCTCCTCCTCCCCCACTTCGGGGCTGGGCTTTTTTTTTTTTTTCGGAGGAGGGGGGGCACAGGACAGCAGCCCCCTTTGCGGGTTGCTCTGGGCTCACTCCAGAGTTCTGTCTGCCTTCCTTCATCCCCCTTTGCCCCGGCCCAGCTCTGGGTCTCACCCCCTACCCCTGTACACCCGTCTCCGGTCTCAGCCCCCTCTGCCAGCCGTGACCAGCCCCCTCCGCTCTCACTGCACTTCTGGCTCGGCCTGTCCTCCCTCTTCCTCTATCTCCCCAAGCTGATCTCTGCTGCTGTCTCTCCA

>Deletion mutation genotyping assay mutant allele amplicon, 330 bp

GGGAAGGGAAGCACTAAGACTGCCCCCAGGGCAGAACCTCGGGGTTTCTGTGCCCCCTCCT▲CCCTGGGCGTGGAGCGGTTTGAGGAGATCCTTCAGGAGGCGGGGTCCCGAGGAGGGGAGGAGCTCGGCCGCAGCTACGGGGAGGAAGACTTTGAATGTGAGGGGGTCGGGGGAGGCGAGTTGGGGGCTGACAGGGTGTTGCTCCGGGCTGGGGGCCTGGGGGCGGGGAGAGGCTGGGGGCCCGCCAGCTGGAGAGGGGGGAGCAGGGCCCATTGGCAGCCAGTGCACCTGTCCCTGCAGACCATCGCCAGTCTTCCCATCACATCCACC

**Figure S1A** displays the bovine *SLC4A2* genomic sequence encompassing the deletion mutation associated with osteopetrosis in Red Angus cattle. Exon sequences are bracketed and highlighted with green bold text. Exons 1-4 are shown, and the start of exon 1 corresponds to the 5’ transcriptional start of GenBank accession number DV927173. Splice donor (**GT**) and acceptor (**AG**) sites are highlighted in black bold text. The start codon within exon 2 is highlighted in red bold text. The 2781-bp deleted sequence is shaded gray, and each breakpoint is marked by a bold double slash (**//**). Sequences denoted with a double strikethrough were identified as repetitive by RepeatMasker. **Figure S1B** indicates the amplicon sequences generated from the PCR-based deletion mutation genotyping assay. Amplicon sizes in base pairs are noted. Primer sites (sense strand) are underlined. The deleted sequence is denoted by the triangle symbol (▲).
